# Identifying barriers and facilitators to COVID-19 vaccination uptake among People Who Use Drugs in Canada: a National Qualitative Study

**DOI:** 10.1186/s12954-023-00826-6

**Published:** 2023-07-29

**Authors:** Farihah Ali, Ashima Kaura, Cayley Russell, Matthew Bonn, Julie Bruneau, Nabarun Dasgupta, Sameer Imtiaz, Valérie Martel-Laferrière, Jürgen Rehm, Rita Shahin, Tara Elton-Marshall

**Affiliations:** 1 Centre for Addiction and Mental Health (CAMH), Institute for Mental Health Policy Research, Toronto, Canada; 2 Ontario CRISM Node Team (OCRINT), IMHPR, Centre for Addiction and Mental Health (CAMH), Room 2035, 33 Russell Street, Toronto, Canada; 3 Canadian Association of People Who Use Drugs, Dartmouth, NS Canada; 4 Toronto Public Health, Toronto, Canada; 5 Centre de Recherche du Centre hospitalier de l’Université de Montréal, Montreal, Canada; 6 Department of Microbiology, Infectious Diseases and Immunology, Université de Montréal, Montreal, Canada; 7 School of Epidemiology and Public Health, Faculty of Medicine, University of Ottawa, Ottawa, Canada; 8 Department of Psychiatry, Dalla Lana School of Public Health, & Institute of Medical Science (IMS), Toronto, Canada; 9 1 King’s College Circle, University of Toronto, Toronto, ON M5S 1A8 Canada; 10 Campbell Family Mental Health Research Institute, Centre for Addiction and Mental Health (CAMH), 1001 Queen St. West, Toronto, ON M6J 1H4 Canada; 11 Institut Für Klinische Psychologie Und Psychotherapie, Technische Universität Dresden, Chemnitzer Str. 46, 01187 Dresden, Germany; 12 Department of Psychiatry and Psychotherapy, Center for Interdisciplinary Addiction Research (ZIS), University Medical Center Hamburg-Eppendorf (UKE), Martinistraße 52, 20246 Hamburg, Germany; 13 Research Centre, Centre Hospitalier de l’Université de Montréal (CRCHUM), 900 Saint-Denis Street, Montreal, QC H2X 0A9 Canada; 14 Department of Family and Emergency Medicine, Faculty of Medicine, Université de Montréal, 2900 Boul, Edouard-Montpetit, Montreal, QC H3T 1J4 Canada; 15 Gillings School of Global Public Health, University of North Carolina, Chapel Hill, NC USA

**Keywords:** COVID-19, Vaccination, People who use drugs, Barriers, Vaccine hesitancy, Vaccine confidence, COVID-19 vaccines, Substance use

## Abstract

**Background:**

People Who Use Drugs (PWUD) have lower vaccination uptake than the general population, and disproportionately experience the burden of harms from vaccine-preventable diseases. We conducted a national qualitative study to: (1) identify the barriers and facilitators to receiving COVID-19 vaccinations among PWUD; and (2) identify interventions to support PWUD in their decision-making.

**Methods:**

Between March and October 2022, semi-structured interviews with PWUD across Canada were conducted. Fully vaccinated (2 or more doses) and partially or unvaccinated (1 dose or less) participants were recruited from a convenience sample to participate in telephone interviews to discuss facilitators, barriers, and concerns about receiving COVID-19 vaccines and subsequent boosters, and ways to address concerns. A total of 78 PWUD participated in the study, with 50 participants being fully vaccinated and 28 participants partially or unvaccinated. Using thematic analysis, interviews were coded based on the capability, opportunity, and motivation-behavior (COM-B) framework.

**Results:**

Many partially or unvaccinated participants reported lacking knowledge about the COVID-19 vaccine, particularly in terms of its usefulness and benefits. Some participants reported lacking knowledge around potential long-term side effects of the vaccine, and the differences of the various vaccine brands. Distrust toward government and healthcare agencies, the unprecedented rapidity of vaccine development and skepticism of vaccine effectiveness were also noted as barriers. Facilitators for vaccination included a desire to protect oneself or others and compliance with government mandates which required individuals to get vaccinated in order to access services, attend work or travel. To improve vaccination uptake, the most trusted and appropriate avenues for vaccination information sharing were identified by participants to be people with lived and living experience with drug use (PWLLE), harm reduction workers, or healthcare providers working within settings commonly visited by PWUD.

**Conclusion:**

PWLLE should be supported to design tailored information to reduce barriers and address mistrust. Resources addressing knowledge gaps should be disseminated in areas and through organizations where PWUD frequently access, such as harm reduction services and social media platforms.

**Supplementary Information:**

The online version contains supplementary material available at 10.1186/s12954-023-00826-6.

## Introduction

As the COVID-19 pandemic continues to evolve, and rates of transmission fluctuate with new variants, vaccination and subsequent boosters have become a primary public health intervention to reduce the severity and spread of COVID-19 [1]. Evidence demonstrates that COVID-19 vaccines can significantly reduce the probability of severe outcomes such as death and hospitalization [1–4]. Although completion of the primary series of vaccination offers some protection, vaccine effectiveness against severe outcomes with additional doses is much higher [3]. Protection offered by this initial dose schedule of the vaccine wanes over time, and booster doses have therefore been recommended to increase the immune response [4].

People Who Use Drugs (PWUD) have intersecting health and social vulnerabilities that elevate their risk for the novel coronavirus disease infection, complications, and mortality [5–8]. PWUD are also at an increased risk of these harms due to social and structural marginalization, such as homelessness and mass incarceration [5, 9] and inadequate access to shelters; and barriers to access for essential harm reduction services (including reduced capacity of services) [8–10]. Sharing used paraphernalia, the inability to self-isolate or socially distance, and housing in crowded shelters or congregate settings may also increase the risk of COVID-19 transmission [9]. PWUD are also at an increased risk for experiencing stigmatization, criminalization, and discrimination, further impeding their access to and trust in formal healthcare [11, 12]. Additionally, evidence has documented that PWUD disproportionately experience harms from, and are more at risk for, infectious diseases such as staphylococcus aureus, HIV, and HCV, and vaccine-preventable infections such as Hepatitis A and influenza due to such socio-structural reasons [13–21]. Combined, these factors highlight the importance of COVID-19 vaccination for PWUD to reduce the likelihood of disease transmission and related adverse outcomes. However, evidence demonstrates that vaccine uptake is low among this population [13, 22, 23]. For example, in Australia, COVID-19 vaccine uptake was lower among PWID compared to the general population in most states and territories (14%-77% versus 39–92%, respectively) [24]. Similarly, in British Columbia, Canada, the general population had higher COVID-19 vaccination rates than PWUD (81% versus 64%, respectively) [25].

While evidence suggests disproportionately lower vaccine uptake among PWUD, few studies have examined barriers and facilitators to COVID-19 vaccination among PWUD, none of which have been conducted within the Canadian context. Three quantitative surveys (conducted in New York City, Oregon, and Melbourne, Australia) and three qualitative studies (conducted in Oregon, rural Illinois, and Philadelphia) were undertaken among PWUD [26–31]. Reasons for getting vaccinated included a desire to protect family and friends [26, 27]. Barriers to vaccination included concerns about safety or side effects of the COVID-19 vaccine [26–30], not being concerned about getting sick from COVID-19 or not being concerned enough to feel that a COVID-19 vaccine is necessary [26, 27, 31], and not feeling like they have enough knowledge about the COVID-19 vaccine [26, 31]. However, these studies were conducted early during the pandemic when vaccines were first being distributed and therefore may not be reflective of ongoing barriers, concerns, and facilitators to vaccination. Accordingly, there is a paucity of research examining recent concerns, barriers and facilitators of the COVID-19 vaccine, and the ways in which concerns related to COVID-19 vaccination uptake among PWUD can be addressed. Additionally, few qualitative studies explored thematic reasons for vaccine hesitancy, and samples have consisted primarily of people who were not vaccinated.

**Fig. 1:**
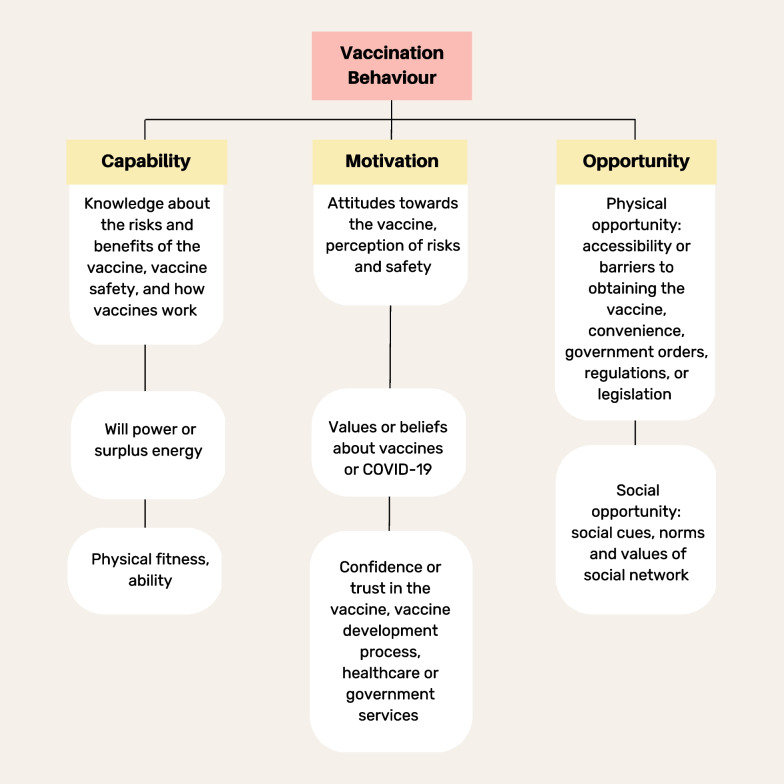
COM-B model as described by The World Health Organization’s (WHO) Tailoring Immunization Program (TIP) [32]

The World Health Organization (WHO) in their Tailoring Immunization Program (TIP) created an evidence-based strategy for developing effective interventions to improve vaccine uptake [32]. The program highlights the importance of engaging stakeholders from the target population to understand drivers and barriers to vaccination [32]. The TIP approach uses a theoretical framework based in psychological research, specifically, the capability, opportunity, and motivation model of behavioral change (COM-B) [32]. TIP suggests that to develop effective interventions, it is necessary to identify capability, opportunity, and motivational factors, as suggested by Fig. 1, that could facilitate or deter vaccination among the population of interest [32, 33]. This model has demonstrated strong predictive value for other behaviors including physical activity, sedentary behavior, and health behavior. For instance, the COM-B framework predicted COVID-19 vaccine acceptability in a study of healthy adults in Iran [34]. This framework has also been applied to understand and explore behavioral predictors of vaccine hesitancy in terms of HPV, MMR (measles-mumps-rubella), and influenza [35–37]. As such, the current study applied the COM-B model to identify barriers and facilitators of vaccination uptake to inform future evidence-based interventions aimed at increasing vaccination uptake among PWUD. The current study builds upon existing research by examining barriers and facilitators to COVID-19 vaccination in a geographically diverse (cross-national) sample of PWUD in Canada.

## Methods

### Study design

The present study was a cross-sectional qualitative study that consisted of one-on-one semi-structured telephone interviews with PWUD from across Canada. The definition of vaccination status for the study was aligned with the Government of Canada Federal regulations at the time of data collection (March 2022–October 2022). This included those who received two doses of a Health Canada authorized vaccine (Moderna, Pfizer-BioNTech, AstraZeneca) in any combination were considered fully vaccinated, or had one dose of Johnson and Johnson, whereby one dose of the vaccine was required to be considered fully vaccinated. Two distinct interview guides were developed, one for fully vaccinated participants and one for unvaccinated or partially vaccinated participants (i.e., those who received no doses at all or have not completed the primary series).

### Eligibility

To be eligible for the study, participants had to have been presently residing in Canada, fluent in English or French, currently using illicit substance(s) at least once weekly and were either fully vaccinated (two or more doses), partially vaccinated (one dose), or unvaccinated (no doses).

### Participant recruitment

Participants were recruited from a pre-existing convenience sample of 200 PWUD that was initially assembled by the Canadian Research Initiative in Substance Misuse (CRISM) Ontario Node, a national research network, for a national qualitative study that occurred in May 2020 regarding the impact of COVID-19 on PWUD’s substance use and access to services, at which time participants agreed to be contacted for future related studies (Research Ethics Board #049/2020) [38]. For the original study, recruitment flyers and posters were distributed on social media and throughout harm reduction and health organization networks in each province and called the toll-free study line or emailed the study email if they were interested in participating. For the present study, an attempt to re-contact these individuals were made via email and telephone, and similar recruitment methods were implemented to recruit additional participants to replenish the sample of individuals who were not able to be re-contacted and were lost to follow-up. Francophone participants were recruited from a pre-existing cohort (HEPCO Cohort) of injection drug users with a history of HIV and/or HCV infection, residing in the greater Montreal area (Research Ethics Board (#20.053) [39]. A French speaking member of our research team at the Centre Hospitalier de l’Universite de Montreal (CHUM) in Quebec contacted participants via telephone, informed them of the study, gauged study interest, and conducted informed consent and the interview with interested participants.

Our aim was to recruit approximately 100 PWUD from across Canada, with the intention of recruiting approximately 50% of participants who were partially vaccinated or unvaccinated.

### Data collection tools and analysis

The interview guides were developed in consultation with the research team, including PWLLE whereby questions focused largely on understanding potential barriers and concerns about receiving vaccinations and ways to address such issues. For vaccinated individuals, questions about their experiences receiving the vaccine were asked, including why they initially decided to get it. Questions also sought to identify those individuals PWUD believed to be the most appropriate and trusted sources to provide information and resources addressing concerns related to vaccination, as well as formats and platforms that would be most beneficial in disseminating such information to reach the PWUD population. Additional file 1 contains copies of the interview guides for both partially and unvaccinated, and vaccinated participants.

Data collection began on March 31, 2022, and ended on October 6, 2022. All interviews were audio recorded for transcription and analysis purposes. Full informed consent was received prior to the interviews. Participants were given $30 honoraria for their time and participation. Francophone interviews were translated into English via a translation company. Once translated, the Francophone interviews were combined with the English transcripts to allow for collaborative analysis. All interviews were transcribed via a third-party translation company and uploaded to NVivo 12 qualitative software for analysis.

An initial codebook of themes was developed and informed by the overarching questions in the interview guides, guided by the COM-B model. Specifically, coders organized responses according to identification of themes around capability, opportunity, and motivation as outlined in the TIPS framework [32]. Capability themes referred to the psychological or physical ability to get vaccinated, opportunity themes referred to the physical and social environments that either facilitated or discouraged vaccination, and motivation referred to the automatic (i.e., impulses and emotions) and reflective processes (i.e., intentions and beliefs) that influenced behavior (see Fig. 1 and WHO, 2019 for further description) [32]. The research team reviewed the codebook in an iterative process as new themes emerged when coding the data. All transcripts were coded by one coder (AK), and 10% were coded by a second coder (CR) to ensure validity of the codes. Discrepancies were reviewed and discussed among coders until agreement was reached. Final themes and results were grouped under the three overarching COM-B concepts, and narratively presented under each below.

## Results

### Sample characteristics

A total of *N* = 114 participants expressed interest in participating; however after screening, 36 were excluded (one participant did not use any substances, four used less than weekly, 23 did not use illicit substances and eight of whom were not able to be reached after initial screening). A total of *n* = 78 PWUD were included in the final study analyses. The average age of participants was 40, with 46% who self-reported as female, 62% who identified their ethnicity as White and 64% who had received 2 or 3 doses of the COVID-19 vaccine (See Table 1 for demographic characteristics).

**Table 1:** Self-reported demographic characteristics of the study participants

Demographic characteristics	Total sample *N* = 78 (%)	Atlantic region *N* = 17 (%)	British Columbia region *N* = 10 (%)	Ontario region *N* = 19 (%)	Prairies region *N* = 14 (%)	Quebec region *N* = 18 (%)
Age (years, mean $\pm$ SD)	40.2 $\pm$ 10.0	31.7 $\pm$ 4.3	36.5 $\pm$ 8.7	44.9 $\pm$ 11.6	34.7 $\pm$ 7.0	50.0 $\pm$ 6.9
Age groups						
18–30	17 (21.8)	6 (35.3)	3 (30.0)	3 (15.8)	4 (28.6)	1 (5.6)
31–50	42 (53.8)	11 (64.7)	6 (60.0)	10 (52.7)	10 (71.4)	5 (27.8)
$\ge 51$	19 (24.4)	0	1 (10.0)	6 (31.6)	0	12 (66.7)
Gender						
Man	42 (53.8)	11 (64.7)	4 (40.0)	9 (47.4)	5 (35.7)	13 (72.2)
Woman	32 (66.7)	5 (29.4)	6 (60.0)	7 (36.8)	9 (64.3)	5 (27.8)
Other	4 (5.1)	1 (5.9)	0	3 (15.8)	0	0
Ethnicity						
White	48 (61.5)	13 (76.5)	6 (60.0)	10 (52.6)	2 (14.3)	17 (94.4)
Indigenous	15 (19.2)	1 (5.9)	2 (20.0)	2 (10.5)	9 (64.3)	1 (5.6)
Black	6 (7.7)	0	1 (10.0)	5 (26.3)	0	0
Other	9 (11.5)	3 (17.6)	1 (10.0)	2 (10.5)	3 (21.4)	0
Remote/rural						
Yes	6 (7.7)	4 (23.5)	1 (10.0)	1(5.3)	0	0
No	72 (92.3)	13 (76.4)	9 (90.0)	18 (94.7)	14	18
Vaccine status						
Vaccinated	50 (64.1)	8 (47.1)	4 (40.0)	14 (73.7)	10 (71.4)	14 (77.8)
Three doses	24 (30.8)	3 (17.6)	1 (10.0)	9 (47.4)	2 (14.3)	9 (50.0)
Two doses	26 (33.3)	5 (29.4)	3 (30.0)	5 (26.3)	8 (57.1)	5 (27.8)
Unvaccinated ….and partially ….vaccinated	28 (35.9)	9 (52.9)	7 (70.0)	5 (26.3)	4 (28.6)	4 (22.2)
One dose	5 (6.4)	2 (11.7)	3 (30.0)	0	0	0
No doses	23 (29.5)	7 (41.1)	3(30.0)	5 (26.3)	4 (28.6)	4 (22.2)

The Atlantic Region includes the provinces of New Brunswick, Newfoundland and Labrador, Nova Scotia, and Prince Edward Island. The Prairies Region includes the provinces of Alberta, Saskatchewan, and Manitoba

In terms of substance use characteristics, most participants endorsed polysubstance use (58%), followed by stimulants (26%) and opioids (12%) (See Table 2 for substance use characteristics).

**Table 2:** Current substance use among study participants

Substance use	Participants *N* = 78 (%)
Substances	
Polysubstance	45 (57.7)
Stimulants	20 (25.6)
Opioids	9 (11.5)
OAT	2 (2.6)
Not specified	2 (2.6)
Frequency of Use	
Daily	42 (53.8)
Weekly	36 (46.2)

‘Stimulants’ primarily included uppers such as cocaine, crack-cocaine, amphetamines and methamphetamine/crystal meth; ‘Opioids’ primarily included downers including both illicit and pharmaceutical opioids such as hydromorphone, heroin, and fentanyl, but excluded references to OAT such as Suboxone or methadone; ‘Polysubstance’ use included reference to using two categories of substances, as well as using speedballs (a combination of stimulants and opioids). ‘Not specified’ included references made by participants to using a variety of unspecified substances

## Narrative results

We have presented our results under the respective conceptual COM-B headings of capability, opportunity, and motivation to address the behavior and uptake of COVID-19 vaccination. The themes are illustrated by select quotes from the interviews, followed by the participants’ vaccination status and geographical location. As not all participants discussed every theme, we have provided the number of participants who endorsed each theme in the narrative results as presented below.

### Capability

Capability refers to whether PWUD have the knowledge, skills, and ability to get vaccinated. Relevant to this study, Capability included PWUD’s knowledge about the COVID-19 vaccine and booster. This section has been organized based on the following subthemes: (1) Lack of knowledge regarding the COVID-19 vaccine and (2) Addressing COVID-19 knowledge gaps.

#### Lack of knowledge regarding the COVID-19 vaccine

When we asked partially and unvaccinated participants what they know about the COVID-19 vaccine, *n* = 15/28 (54%) participants reported not having the knowledge or understanding of the vaccine in terms of its usefulness and benefits:

What do I know about it? I know that a lot of people don’t know anything about it. About any of the vaccines that people are getting lately. (British Columbia, Unvaccinated)

Lacking knowledge around potential long-term side effects of the vaccine was reported (*n* = 23/78; 29%) as a concern among unvaccinated, partially vaccinated, and vaccinated participants. Some participants expressed wariness around the vaccine due to not knowing how it will affect them in the future:

Well, the reason why I haven’t got it [COVID-19 vaccine] is because nobody knows any long-term effects. The testing hasn’t been done for long-term effects, why would I let it [COVID-19 vaccine] be in my body when it’s [COVID-19] not gonna kill me anyways (Nova Scotia, Unvaccinated)

This was further expressed by the following participant, who elaborated on the unknown effects:

It [the vaccine] could have been something that the human body would have badly reacted to that, and I don’t know, pimples growing on your face or things like that. We don’t know. It’s not a vaccine that was tested long term so we didn’t know much about if it would have side effects or not (Quebec, Vaccinated)

Some participants (*n* = 12/78; 15%) noted that they did not have sufficient information regarding the differences or effects of the various vaccine brands. Others (*n* = 20/78; 26%) expressed preference for certain COVID-19 vaccine brands due to the information they had received:

Yeah, I was hoping for Moderna and that’s what I did get. I definitely didn’t want the AstraZeneca and was trying to figure out which one was better and Moderna was more all-encompassing, so I was hoping for Moderna and that’s what I ended up getting. (New Brunswick, Vaccinated)

*N* = 26/78 (33%) participants described a lack of information regarding the impacts of mixing vaccine brands and indicated this lack of information made them hesitant to receive a booster of a vaccine that was a different brand than their first dose. In some instances (*n* = 4/26; 15%) this lack of knowledge deterred participants who had received two doses from receiving a booster.

#### Addressing COVID-19 knowledge gaps

To address overall concerns related to the lack of knowledge, 71% (*n* = 20/28) of unvaccinated and partially vaccinated participants noted that receiving information related to the side effects, details on how the COVID-19 vaccines worked and were developed, as well as the usefulness of receiving the vaccine, would make them feel more comfortable to get vaccinated:

Learning more about it [COVID-19 vaccine], like how they got the antibodies and stuff like that. Or how it’s reacting inside the body and if it’s not doing any harm or if it’s changing any sort of organs or cells or things like that. (Alberta, partially vaccinated)

To ensure information is accessible, all participants were asked what formats or platforms they would find information related to COVID-19 as the most accessible for hosting information about the vaccines and boosters. Several easy-to-understand formats were suggested, including pamphlets (*n* = 10/78; 13%), posters (*n* = 6/78; 8%), and advertisements (*n* = 6/78; 8%). Regarding the platforms to reach PWUD, social media (*n* = 22/78; 28%), news channels and television networks (*n* = 17/78; 22%), and harm reduction facilities (*n* = 20/78; 26%) were identified as the most accessible to PWUD and were settings PWUD would access. Some participants (*n* = 3/78; 4%) also mentioned including information about vaccinations in safer drug use kits which are commonly accessed by PWUD:

I think you could put you know, when you’re giving out safe using supplies you can put out a little bit of information on where to get a vaccine, why you should get one, things like that. Reasons that you can trust the government on this issue. (Nova Scotia, Vaccinated)

PWLLE, healthcare workers, and peer support or harm reduction workers were cited by participants as the most appropriate and trusted individuals to provide information and resources about vaccinations. One participant discussed how having a PWLLE share their experience about getting the vaccine could be a positive way to encourage others to get vaccinated:

If you’re targeting people with addiction for it [the vaccine] then maybe some peers with lived experience and stuff like that so they feel comfortable, like, oh hey, this person has gotten the vaccine and they’re fine, they’re an active user and were at the time and it didn’t affect their heart or their use, like you know, people are nervous about it. (Newfoundland, Vaccinated)

Overall, this section illustrates a gap in knowledge about the COVID-19 vaccine among PWUD. Participants suggested that receiving information regarding the vaccine development process, the side effects, as well as the usefulness and efficacy in easy-to-understand formats, such as posters and pamphlets, would increase their knowledge about the vaccine. Furthermore, PWLLE were identified as being the most trustworthy source for sharing such information and increasing PWUD confidence in the vaccine and subsequent boosters, in addition to other trusted healthcare professionals that work closely with PWUD.

### Opportunity

The opportunity theme refers to the physical and social contextual environment that either facilitates or discourages vaccination. This section has been divided into two subthemes: (1) Physical environment, including logistical barriers and government mandates, and (2) Social environment, including social norms related to vaccination uptake among participant networks.

#### Physical environment

##### Logistical barriers

Factors in the physical environment which may encourage or discourage vaccination as described by participants included access to and availability of vaccination services, and the appeal of vaccines among PWUD.

Some vaccinated participants (*n* = 14/50; 28%) discussed logistical issues when asked about the convenience and accessibility of obtaining the vaccine. Booking appointments via website portals (*n* = 9/14; 64%), difficulty finding or paying for transportation to get to vaccination clinics (*n* = 2/14; 14%), wait-times to book an appointment (*n* = 6/14; 43%), and for some, not having appropriate identification to receive the vaccination upon arrival at the clinic (*n* = 3/14; 21%) were identified. These issues were not mutually exclusive, and were discussed as barriers to PWUD’s convenient access to getting the vaccine, as illustrated by the following quote:

There were times where there were no appointments, and it was fully booked. So, it did take a lot of checking the website consistently to find an appointment. (Nova Scotia, Vaccinated)

The systems were very hard to set up and not a lot of times, you know, carrying around the card [ID]. I’m a drug user, I lost my ID. I still don’t have an ID, I had to use a picture off my phone. So that’s really hard. (Nova Scotia, Vaccinated)

Other barriers identified by vaccinated participants included a fear of needles (*n* = 2/50, 4%) and distance to travel to the vaccination clinic (*n* = 3/50, 6%).

However, these barriers did not prevent participants from obtaining the vaccine, and 58% of vaccinated participants (*n* = 34/50) identified no barriers in the process of obtaining the vaccine:

It’s easy accessing one [vaccine], so I don’t think there are barriers to accessing one. (New Brunswick, Vaccinated)

Two unvaccinated or partially vaccinated participants discussed how they were feeling overwhelmed with their substance use, which took priority over getting vaccinated:

"It’s almost like the space in my head has been taken up by that – By the issue of the drug use, that having the time to think about some of those things is difficult, having the money to either drive or take a bus or go somewhere and then dealing with appointments, etcetera, it definitely affects that. It’s sort of hard to be motivated (British Columbia, partially vaccinated)

##### Government mandates

Physical opportunity also encompasses legislation and regulations that promote vaccination uptake. Unique to COVID-19, government mandates in most provinces of Canada enforced proof of vaccination requirements, which required individuals to vaccinate in order to travel, and depending on provincial regulations, sit in at restaurants, access facilities or services and remain employed. 38% (*n* = 19/50) of vaccinated participants described feeling forced to obtain the vaccine due to such mandates, as described by the following participants:

I decided [to get vaccinated] because I was stuck in my house for so long and it was just driving me crazy and I just needed to get out and I just had no choice but to get vaccinated…No, I didn’t wanna get it at all. I didn’t want it at all. I just felt like I was forced to so I could go out. (Manitoba, Vaccinated)

This was also relevant for participants who were mandated to get vaccinated as part of their employment:

I work on the frontlines in harm reduction, and they take it serious among the harm reduction program managers and I wanted to keep working and that was one of the stipulations of being on the frontline. (Nova Scotia, Vaccinated)

#### Social environment

Social opportunity addresses factors in the social environment including whether vaccination is a social norm in the participant’s community, as well as whether their peers and family members were vaccinated.

##### Family and peer network influence on COVID-19 vaccination

To understand vaccination uptake relevant to PWUD social networks, unvaccinated and partially vaccinated participants were asked whether people close to them such as their family and friends had been vaccinated. Many participants *n* = 15/27 (56%) reported that people within their social network have been vaccinated, primarily due to mandates, while *n* = 5/27 (19%) reported that their social network was unvaccinated, and *n* = 7/27 (26%) reported a mix of their social network being both vaccinated and unvaccinated.

Overall, the opportunity section illustrates that while some logistical barriers were experienced by vaccinated participants when obtaining their vaccination, it did not prevent them from getting vaccinated. The provincial mandates acted as a major driving force for some vaccinated participants, who reluctantly got their vaccine due to these measures. Furthermore, when exploring social norms within PWUD regarding vaccination uptake, unvaccinated and partially vaccinated participants noted mixed responses on whether their social networks were vaccinated.

### Motivation

Motivation relates to automatic (i.e., impulses and emotions) and reflective processes (i.e., intentions and beliefs) that can impact COVID-19 vaccine uptake. Within this theme, participants’ attitudes, perceptions, and risk assessment of the vaccine and COVID-19; intention to be fully vaccinated; fears and concerns about vaccine safety or COVID-19; and trust in healthcare agencies and workers were explored. These themes are illustrated by the following subthemes: (1) Self-protection and protecting others, (2) Vaccine-related concerns, (3) Structural distrust against the government and healthcare systems, and (4) Concerns related to booster doses.

#### Feelings of self-protection and protecting others

Among vaccinated participants, one of the most common reasons as to why they obtained the vaccine and subsequent boosters was due to self-protection and protecting others (*n* = 30/50; 60%). The following participant expressed that they decided to get vaccinated to protect his elderly father who may be more susceptible to COVID-19:

Because I wanted to avoid contaminating people, mainly my father who has a weak system and in poor shape and older. That was the first reason maybe. To protect him and to do my civic duty also (Quebec, Vaccinated)

Another participant articulated how they were working on the frontlines and in high-risk environments, and as such, needed to get vaccinated to protect themself:

I worked doing outreach and was coming into contact with hundreds of people in a week and there was very little protection even with PPE (personal protective equipment), it was close quarters especially when working outside and we didn’t have a lot of control over the environment, and I was at pretty high risk. (British Columbia, Vaccinated)

*N* = 9/30 (30%) of vaccinated participants who said they got vaccinated for protection further articulated that they trusted the scientific knowledge of the COVID-19 vaccine and information disseminated regarding the importance of getting vaccinated as a public health measure to reduce their risks of contracting and spreading COVID-19.

#### Vaccine-related concerns

When unvaccinated and partially vaccinated participants were asked whether they thought the COVID-19 vaccine was useful, *n* = 21/28 (75%) of participants suggested that receiving the COVID-19 vaccine was not useful to them, particularly because they believed they were healthy enough to withstand the COVID-19 virus (*n* = 10/28, 36%). This was illustrated by the following participant who mentioned they had a strong immune system, and had never contracted COVID before, iterating their belief that the vaccine would not be of use to them:

Basically, because my friends and family are all vaccinated and the fact that I’ve had COVID tests and they’ve all come up negative, and I don’t have serious health issues or lung issues. I think I’d be able to ward it off okay. I have a strong immune system. (New Brunswick, Unvaccinated)

These sentiments were also emphasized by one participant who likened the COVID-19 virus to the flu and discussed how they never got the flu vaccine and had never contracted the flu, believing that they are healthy enough to also withstand getting COVID without getting the vaccine:

I mean I don’t get my flu vaccine and in my eyes that’s what it is, a flu shot. And I haven’t been one to get the flu vaccine and I’m pretty good at keeping myself healthy, so I don’t see any benefit. (Ontario, Unvaccinated)

In addition, 14% (*n* = 11/78) of participants questioned the effectiveness of the vaccine, expressing concerns about acquiring the virus despite being vaccinated. This was described by one participant who expressed confusion about the benefit of the vaccine as people they knew had gotten the vaccine but had still contracted COVID.

Well, first the effectiveness. Because, like, I knew people that had got the vaccine and got COVID, so there was that piece. It was kind of like, is it even working and what is it if it’s not working, like what is it doing to me? (Ontario, Vaccinated)

Participants also expressed fear and concern about the perceived speed of vaccine development and side effects (*n* = 47/78; 60%). Vaccinated, unvaccinated and partially vaccinated participants (*n* = 27/78; 35%) articulated that due to the expedited nature in which the vaccine was developed, they were concerned about the safety and related harms:

I just think that it [COVID-19 vaccine] was really rushed to be put out to the public. There wasn’t a lot of time, vaccines take years to put together and even then they’re not full of the best ingredients…it seemed super sketchy to me, people are just kind of blindly following along. And just felt compelled to get the vaccine. (Ontario, Unvaccinated)

Overall, concerns related to the vaccine development, effectiveness and usefulness were discussed as lack of motivating factors which impacted some PWUD from getting the vaccine.

#### Structural distrust against government and healthcare systems

Structural concerns such as distrust in the government and healthcare system in the context of being a PWUD were brought up by *n* = 27/78 (35%) participants as factors which may impact their decisions to get vaccinated. The historical distrust against and negative experiences with the medical system was discussed by the following participant:

Well it’s just that we’re over-researched, we’re not treated properly within the community and the government. They don’t see people who use drugs the same. So I don’t know, I just feel like some of that stigma and all that societal pressure from being a drug user like makes you not trust the medical system because they treat us like shit every day when we go to the hospital. So it makes us not trust them, so I think that added like another layer of not wanting to do it [get vaccinated] (Ontario, Vaccinated)

Similarly, another participant discussed how situations that directly impact them such as the opioid crisis have been conveyed to the public in untruthful ways. The participant discussed frustration that the government’s response to the COVID-19 pandemic was mobilized rapidly, while responses to the opioid crisis have been slow, despite the associated harms being longstanding and well-documented:

Just with COVID and everything there was a lot of conspiracy theories going on and different information from all kinds of different sources and it was really hard to get a gauge on what was true. Especially as a drug user and someone who has seen constant lies from the government about the ongoing opiate epidemic and toxic drug poisoning crisis that the announcements that they were making didn’t land in any kind of truthful way. And also it seemed incredibly hypocritical that they can pull out this mass mobilization in such a short period of time and we’re within year six of the overdose crisis and have barely moved an inch. (British Columbia, Vaccinated)

Fifty percent (*n* = 14/28) of unvaccinated and partially vaccinated participants also reported having doubts about the legitimacy of the vaccine, and identified conspiracy-related theories, stemming from systematic distrust against the government and medical system, as a reason to not get vaccinated:

I kind of had that worry about the population control, like you know, they could be injecting anything into us. Like we don’t know what’s going on in the upper government, and stuff like that. You know. They could be injecting something for population control (Nova Scotia, Unvaccinated)

One participant reported being vaccinated without permission in the hospital, which contributed to distrust toward the medical system and healthcare agencies. This participant reported having to go to the hospital for an unrelated issue and being vaccinated due to being in the Intensive Care Unit (ICU):

I didn’t get my shot until December when I was in the hospital, I was in the ICU (intensive care unit), and they gave me the shot because they had to be around me, so they gave me that shot even though I tested negative. I had a blood infection, it had nothing to do with COVID. But they still gave it to me....And they said, oh well she doesn’t have a COVID shot right? And my daughter said no, she doesn’t, and they said well, we’re giving her first COVID shot then. There’s a lot of people around here with COVID and we don’t want her to get it. (British Columbia, Partially Vaccinated)

#### Booster doses

Motivation for obtaining the booster dose was explored for those participants who had received two doses of the vaccine (*n* = 26/78; 33%). Over half (*n* = 15/26; 57%) of vaccinated participants with two doses of the vaccine reported that they were not interested in getting the booster dose, while other vaccinated participants with two doses (*n* = 11/26; 42%) were considering it. Of those who were not considering the booster, some (*n* = 6/15; 43%) cited frustration with having to obtain multiple vaccines to attain desired protection and others (*n* = 9/15; 64%) mentioned concerns related to the usefulness or effectiveness of the booster. The following participant expressed confusion and frustration with the number of required doses:

I just think there’s too many [doses], like even having a second dose of the vaccine was too much for me. And now they want me to booster in my arm? I’m not with it. (Winnipeg, Vaccinated)

This concern was also discussed by another participant who mentioned they had gotten two doses of the vaccine, yet still contracted COVID, and stated that they would be better off being more diligent with handwashing and wearing masks than getting a subsequent booster:

I had the vaccine I was less careful with my PPE, and that’s when I got it. And I still caught it even though I had this magical vaccine. So that makes me think I’m safer about being careful about handwashing and masks and things like that when I’m at work than getting a new round of chemicals pumped into me (British Columbia, Vaccinated)

Those who were still considering getting their third booster dose explained that they had not received it yet due to a variety of reasons including: not having enough time, not being able to access their vaccine brand preference, not being eligible yet due to public health recommendations of having to wait a particular time period before being eligible, or lacking information around boosters and their effectiveness and purpose.

For five participants (*n* = 5/78; 6%) who had obtained one dose of the vaccine, motivation for obtaining the second dose of the vaccine was explored. Each participant described a different reason for why they had not obtained the second dose, including being overwhelmed by mental health and substance use issues, perceiving the vaccine to not be useful, and general uncertainty about the vaccines. One participant reported receiving a financial incentive for the first dose and did not get the second due to mandates being lifted.

The motivation section illustrates that some vaccinated participants trusted the scientific knowledge of the vaccine and its usefulness which motivated them to get vaccinated. However, for other participants, vaccine-related concerns such as perceptions of the lack of effectiveness and usefulness of the vaccine were discussed as discouraging them from getting vaccinated and subsequent boosters. Structural distrust with the healthcare system and government were also recognized by some participants which contributed to their lack of trust with such systems.

## Discussion

The current study qualitatively identified key barriers and concerns related to COVID-19 vaccination uptake among a national convenience sample of PWUD, a population that is at an elevated risk for contracting COVID-19 infection and experiencing complications [40, 41] and mortality [42]. The results provide insight into how capability, opportunity, and motivation can be addressed with tailored interventions to drive vaccination uptake. Addressing knowledge gaps about the COVID-19 vaccine, its effects, and purpose can improve vaccination capability among PWUD, while highlighting the vaccine’s protective effects in relation to the self and others, and alleviating vaccine-related concerns, can improve motivation.

Consistent with previous studies regarding COVID-19 and other vaccinations, our results demonstrated that PWUD were concerned about the speed of vaccine development, safety of use, side effects, usefulness, and experienced distrust toward healthcare and government agencies involved in vaccine distribution [26–31, 43]. We found that many unvaccinated and partially vaccinated participants reported a lack of knowledge about the COVID-19 vaccine, which discouraged vaccine uptake and amplified already existing medical and government mistrust within this community. Some unvaccinated PWUD additionally reported feeling that they were healthy enough to withstand the virus without the vaccine, which can be contributed to a lack of education or knowledge about the effectiveness of the vaccine and the risks of contracting the virus. Research among other populations that face marginalization such as racialized and Indigenous peoples [44, 45], individuals from low-income households [46], those living in rural areas [47], and those experiencing housing insecurity [48] further corroborate our results. This data suggests that distrust of governments and public health agencies and knowledge barriers can contribute to disparities in vaccine uptake.

The study also highlights the importance of capability factors in contributing to vaccination. Many individuals in our study suggested that vaccines were easy to obtain and indicated that this was an important facilitator to vaccination. Government mandates also contributed as a motivator to vaccination. While government mandates are not unique to Canada, these measures were identified as facilitators which encouraged vaccination among some PWUD in our study, some of whom were vaccinated despite feeling reluctant in order to adhere to these measures. Other studies have also identified that government mandates and proof of vaccination requirements have a statistically significant impact on COVID-19 vaccination [49, 50]. However, compulsory regulations do not necessarily address medical distrust, knowledge gaps regarding the vaccine, or concerns about its side effects, usefulness, or effectiveness. They may also result in the unintended consequence of further entrenching government distrust. Thus, regardless of mandates and ample opportunity as described under the COM-B framework, low capability and motivation play a large role in whether PWUD decide to obtain the vaccine.

The themes identified in the current study have important implications for guiding interventions to increase vaccine uptake among PWUD. There is some research suggesting that addressing concerns about the speed of vaccination development and the safety of the vaccine and highlighting personal benefits of vaccination can be effective strategies to address hesitancy among individuals who are strongly hesitant [51].

In terms of the format for such interventions, informational resources addressing aforementioned concerns should be plain-language, easily accessible, and succinctly described. Evidence demonstrates that writing in plain language, including using minimal or no scientific terminology, brief sentences, and a conversational tone, improves the quality and accessibility of resources [52]. Our findings suggest that this information should be distributed by peers, in settings and platforms commonly accessed by PWUD, such as harm reduction services or social media. In particular, hearing about the benefits of the vaccine and related experiences from peers and others PWUD trust was suggested as a key way to reduce hesitancy and increase knowledge. This highlights the important role of peer modeling to improve capability and motivation in PWUD. Given issues of trust with public health and government, PWUD can play an important role in co-designing and delivering resources to address vaccine hesitancy among PWUD. This is also recognized in other research pertaining to vaccine hesitancy among PWUD, which suggests that public health outreach led by trusted peers and within community harm reduction settings such as needle exchange programs will likely promote COVID-19 vaccination uptake among PWUD [44, 52–55]. Other research that has identified trustworthy sources as mechanisms in disseminating COVID-19 vaccine information included peer recovery support specialists, particularly those who partnered with someone in healthcare [27]. In recognizing inherent distrust among PWUD and the healthcare system and government, having peers who they trust and share commonalities with (or other health professionals who they have an established relationship with) share knowledge on the vaccine would be an effective facilitator to reduce concerns.

Trusted individuals such as healthcare and service providers can also play an important role in addressing capability, opportunity and motivational factors through engaging in motivational interviewing (MI). MI has been identified as an effective strategy in changing behaviors related to vaccination uptake among both marginalized and nonmarginalized communities [56]. MI is defined as a brief conversational approach that seeks to enhance an individual’s motivation for behavioral change [57]. It is participant-centered and goal-oriented, focusing on exploring individuals own desire, ability, and reasons for change. MI was originally developed to treat addictive behaviors and has been shown to be successful in addressing illicit substance use [57, 58], inferring that it may be an appropriate strategy in working with PWUD. MI has also been used among several populations that face marginalization including HIV-positive [59], racialized [60, 61], and women involved within the criminal justice system [62]. MI has led to increases in children’s vaccine coverage [64], human papillomavirus (HPV) vaccination rates among adolescents [65], influenza vaccination rates among college students [64], hepatitis B vaccinations among adults with diabetes [66], among others. Relevant to this study, MI among healthcare professionals, including peers who work directly with PWUD could involve asking open-ended questions to understand and explore PWUD’s concerns about the vaccine and reasons for hesitancy, responding with empathy and validation, providing information with permission about the vaccine to address these concerns, and discussing next steps to help facilitate access.

Other strategies, such as multicomponent interventions, were identified as effective strategies in promoting vaccine uptake [67]. This included dialogue and plain-language informational resources. Thus, to help facilitate COVID-19 vaccination uptake among PWUD, a combination of MI interventions coupled with plain language, accessible resources could be effective in facilitating vaccination uptake.

Our study also found that many respondents did not feel motivated to obtain a booster dose in the future. Individuals were concerned about the usefulness of the booster and were frustrated about the need for ongoing vaccination. This suggests that in addition to developing strategies to address vaccine hesitancy, it will also be important to develop strategies to promote booster vaccinations among PWUD.

## Limitations

It is important to note that PWUD are not a homogenous group and may have additional concerns (intersectionality or differences in experiences according to types and patterns of substance use and other unique personal characteristics) that were beyond the scope of the current study given the small sample size. However, despite the heterogeneity, many common themes were noted, as we reached data saturation. Given that some of the interviews were conducted among individuals who had previously responded to a study on COVID-19, the sample may be over-represented by individuals who are more likely to participate in studies and therefore may not be representative of the general population of PWUD. Interviews were also conducted by telephone, which may exclude marginalized PWUD (including individuals who are experiencing homelessness in particular) who do not have access to this resource. Additionally, while we initially intended to recruit a sample of 50% unvaccinated PWUD, by the launch of the study the rollout of government mandates, vaccinations, and subsequent boosters were well underway, and this was more challenging. However, we were able to explore barriers and facilitators to vaccination and to receiving vaccine boosters among those vaccinated and reached data saturation.

## Conclusions

The study demonstrates several concerns PWUD have regarding the COVID-19 vaccination in Canada. A lack of knowledge about the COVID-19 vaccines, including their usefulness, effectiveness, side-effects, and uncertainty around the development process, were noted as key barriers for many PWUD. These factors are important to consider and address when developing interventions to support vaccine uptake. Concerted efforts must be made to ensure interventions and materials are developed in ways which address the concerns identified by PWUD to improve vaccination uptake. Public health strategies led by peers within settings and organizations which are frequently accessed by PWUD, such as harm reduction settings or social media, can help improve uptake. Ensuring that interventions are developed to address capability, motivation, and opportunity among PWUD can help improve vaccination uptake and behavior in meaningful and appropriate ways. This study has important implications for COVID-19 and subsequent vaccination boosters and can be used to inform future vaccination strategies among this population.

## Supplementary Information

**Additional file 1.** Interview guides for both partially and unvaccinated, and vaccinated participants.

## Data Availability

The datasets used and/or analyzed during the current study are available from the corresponding author on reasonable request.

## Abbreviation

PWUD: People who use drugs
COM-B: Capability, opportunity, motivation model for behavioral change
MI: Motivational interviewing
PWLLE: People with lived and living experience
