# Supplementary material for: Identifying barriers and facilitators to COVID-19 vaccination uptake among People Who Use Drugs in Canada: a National Qualitative Study
Source: Harm Reduct J. 2023 Jul 29;20:99. doi: 10.1186/s12954-023-00826-6 (PMC10387201; doi:10.1186/s12954-023-00826-6)
Supplement: Supplementary file 1 — Additional file 1. Interview guides for both partially and unvaccinated, and vaccinated participants. [file 12954_2023_826_MOESM1_ESM.pdf]

INTERVIEW GUIDE

Interview Guide for Unvaccinated and Partially Vaccinated Participants

1. Can you please confirm your vaccination status?
2. Generally speaking, is there anything about COVID-19 that you are worried about?
   PROBE: Are/were you worried about getting COVID-19? Has anyone you know got COVID-19? Do you follow public health measures such as practice social distancing, wearing a mask etc.?
3. Generally speaking, what do you know about the COVID-19 vaccine?
   PROBE: Where and from whom did you get this information from?
   <interviewer note: probe for understanding relationship with the person who gives them the information>
   PROBE: Do you see any benefits of getting the COVID-19 vaccine?
4. Have people close to you such as friends, colleagues and/or family gotten the COVID-19 vaccine?
   PROBE (if some of the people close to them have not been vaccinated): What are some of their concerns about getting the COVID-19 vaccine?
   PROBE (if people around them have been vaccinated): What, if you are aware, was the reasoning for them to get vaccinated?
5. What are your concerns about getting the COVID-19 vaccine?
   PROBE: Are any of these concerns specific to your drug use?
   PROBE: Are there any barriers that people are likely to face getting vaccinated?
   PROBE: Are these concerns related to specific vaccines (Moderna, Pfizer, AstraZeneca), or vaccines in general?
6. What, if anything, would make you feel comfortable about getting the COVID-19 vaccine?
   PROBE: Is there specific information you would like to know about the vaccine?
   PROBE: Is there a specific place and/or a specific person that you would feel comfortable getting the vaccine from (e.g. harm reduction site such as Needle and Syringe program or overdose preventions site worker)?
7. If any concerns you had related to the COVID-19 vaccine were addressed, how likely would it be for you to get the vaccine?
8. What are the best ways to give people information about the COVID-19 vaccine?
   PROBE: Who would you trust to give you information about the COVID-19 vaccine?
   PROBE: In what format(s) should this information be given? (e.g. storytelling, comic books, online, booklets etc.)
   PROBE: Which locations should this information be distributed? (e.g. online vs. in person, organizations like CAPUD? Harm reduction groups? Community health centres?)
9. Is there anything else you would like to discuss related to the COVID-19 vaccine?

Interview guide for Vaccinated Participants

1. Can you please confirm your vaccination status?
2. Why did you decide to get vaccinated for COVID-19?
   PROBE: What concerns, if any, did you have before you got vaccinated for COVID-19?
   PROBE: Were concerns different depending on the vaccine type (ie. Pfizer, Moderna, Astra Zeneca)?
   PROBE: Did any public health measures encourage you to get it?
   PROBE: How were your concerns addressed?
3. Can you please tell me about the process in which you got vaccinated (signed up, someone facilitated it for you etc.)
   PROBE: Where did you get vaccinated each time (mass vaccination clinic, drop-in centre, harm reduction site, doctor's office)?
   PROBE: Did you experience any barriers or difficulties to getting vaccinated?
4. How likely are you to get a booster shot (if haven't already)?
   PROBE: If have received the booster, how were there experiences the same/different?
   PROBE: If haven't received the booster, did you try to get the booster vaccine and were unable to? What got in the way? What was the experience like?
5. What are some of the biggest concerns your peers may have about a COVID-19 vaccine?
   PROBE: How do you think these concerns can be addressed?
6. Was there anything that helped you in your decision to get vaccinated for COVID-19?
7. PROBE: What information sources do you trust on COVID?
8. PROBE: Who within your social network do you turn to when you have questions about COVID? Why do you trust them?
9. What messages are needed to inform people about the benefits of getting vaccinated?
10. What are the best ways to give people information about the COVID-19 vaccine?
    PROBE: How willing would you be to share information with networks of other PWUD about getting the COVID-19 vaccine?
    PROBE: Who would you trust to give you information about the COVID-19 vaccine?
    PROBE: In what format(s) should this information be given? (e.g. storytelling, comic books, online, booklets etc.)
    PROBE: Which locations should this information be distributed? (e.g. online vs. in person, organizations like CAPUD? Harm reduction groups?)
11. Is there anything else you would like to discuss related to the COVID-19 vaccine?
